# Correlational Analysis of Liver Metabolites and Pharmacodynamic Indexes in Xanthoxylin-Treated Acute Liver Failure

**DOI:** 10.3390/molecules31132231

**Published:** 2026-06-24

**Authors:** Fengfeng Xie, Huimin Luo, Yuchen Shen, Xiuqi Yu, Dudong Wei, Liba Xu, Hua Zhu

**Affiliations:** Guangxi Key Laboratory of Zhuang and Yao Ethnic Medicine/University Engineering Research Center of Development and Industrialization of Zhuang and Yao Ethnic Medicinal Materials, Guangxi/Guangxi Engineering Research Center of Ethnic Medicine Resources and Application, Guangxi University of Chinese Medicine, Nanning 530200, China; 15177143553@163.com (F.X.);

**Keywords:** xanthoxylin, acute liver failure, NF-κB signaling pathway, metabolites, inflammatory factors

## Abstract

Acute liver failure (ALF) is characterized by a rapid decline in liver function, leading to metabolic and organ failure. This study employed liver metabolomics, Nuclear Factor kappa-B (NF-κB) signaling pathway analysis, and inflammatory factor profiling to investigate the therapeutic mechanisms of xanthoxylin in ALF. Xanthoxylin administration led to increased antioxidant levels and reduced markers of inflammation and tissue damage. Xanthoxylin downregulated the messenger RNA (mRNA) expression of Nitric Oxide Synthase (*NOS*), Interleukin-1β (IL-1β), Interleukin-6 (IL-6), Tumor Necrosis Factor-α (*TNF-α*), *NF-κB*, Inhibitor of NF-κB α (*IκBα*), and Toll-like receptor 4 (*TLR4*), and inhibited the protein expression of p-p38 and p-p65 while upregulating B-cell CLL/Lymphoma 2 (Bcl-2) and B-cell Lymphoma-x (Bcl-xl). Metabolomic analysis identified 41 differentially expressed metabolites, 20 of which showed strong correlations with pharmacodynamic parameters. These 20 candidate metabolite signatures are involved in amino acid and carboxylic acid metabolic pathways, with potential links to glycolysis and the tricarboxylic acid (TCA) cycle. Together, these findings suggest that xanthoxylin exerts therapeutic effects against ALF by modulating the IκBα/NF-κB signaling pathway and related metabolic pathways, providing a scientific basis for understanding its multi-target mechanism.

## 1. Introduction

ALF is a critical clinical syndrome characterized by the rapid deterioration of hepatic function, leading to significant metabolic disturbances and multi-organ dysfunction [1]. The incidence of ALF is on the rise, attributable to diverse etiologies such as drug-induced liver injury, viral hepatitis, and metabolic disorders. Current research underscores the pivotal roles of oxidative stress, inflammation, and apoptosis in the pathogenesis of ALF [2]. For instance, research on fisetin has demonstrated its protective effects against ischemia–reperfusion-induced liver damage by reducing oxidative stress markers and enhancing antioxidant capacity, indicating its potential for alleviating liver injury [3]. Accumulating evidence suggests that the TLR4/NF-κB pathway is crucial for the inflammatory response in ALF, as TLR4 activation by lipopolysaccharide (LPS) and danger signals triggers NF-κB activation and pro-inflammatory cytokine production, thereby exacerbating liver injury. Targeting this pathway attenuates liver damage, as calycosin has been shown to inhibit hepatocyte apoptosis by suppressing TLR4/NF-κB signaling [4]. Furthermore, the interaction among pathways is evident in liver injury models. Liensinine, which exhibits anti-inflammatory properties, alleviates sepsis-induced liver injury by inhibiting NF-κB and Mitogen-Activated Protein Kinase (MAPK) pathways via Nuclear factor Erythroid 2-related Factor 2 (Nrf2). This suggests targeting these pathways can mitigate inflammation and oxidative stress in liver injury [5]. ALF involves excessive inflammatory cytokines like TNF-α and IL-6, which induce hepatocyte damage and systemic inflammation. TNF-α, which is released early in liver injury, is associated with NF-κB signaling [6]. Investigating these mechanisms may facilitate the development of improved therapeutic strategies for ALF and its complications. In addition, the balance among Bax, Bcl-2, and Bcl-xL plays a key regulatory role in ALF. Under normal conditions, the anti-apoptotic proteins Bcl-2 and Bcl-xL protect hepatocytes by maintaining mitochondrial membrane stability [7]. During ALF, the pro-apoptotic protein Bax is upregulated, while the anti-apoptotic protein Bcl-xL is downregulated, resulting in an increased Bax/Bcl-xL ratio. This promotes mitochondrial outer membrane permeabilization and the release of cytochrome c, which subsequently activates the caspase cascade, ultimately inducing extensive hepatocyte apoptosis and exacerbating liver failure [8]. Given the role of the TLR4/NF-κB signaling pathway in ALF, as well as the known anti-apoptotic functions of Bcl-2 family proteins in ALF, we selected these targets for hypothesis-driven validation.

Metabolomics technology serves as a valuable tool for studying ALF, facilitating in-depth elucidation of its metabolic changes. An Nuclear Magnetic Resonance (NMR)-based serum metabolomics study found significant metabolic changes in patients with acute-on-chronic liver failure (ACLF) from alcoholic liver disease, with reduced amino acid and lipid metabolites proposed as putative biomarkers for diagnosing and monitoring ACLF [9]. High-throughput metabolomics with Liquid Chromatography-Mass Spectrometry (LC-MS) has been employed to characterize metabolic changes in alcohol-induced liver injury, leading to the identification of potential therapeutic targets for alcoholic liver disease [10]. Collectively, these findings reveal that the identification of critical metabolic alterations and potential biomarkers may enhance diagnostic and therapeutic strategies for acute liver failure, thereby improving treatment outcomes.

Xanthoxylin is a natural compound with a rigid ring structure in its molecular architecture and multiple hydroxyl groups, which endow it with antioxidant, anti-inflammatory, and anti-tumor properties [11]. Xanthoxylin alleviates the inflammatory response by inhibiting the release of pro-inflammatory cytokines and the activation of the NF-κB signaling pathway [12]. Additionally, xanthoxylin exerts a prominent inhibitory effect on human hepatocellular carcinoma Human Hepatocellular carcinoma Grade 2 (HepG2) cells, with its mechanism of action involving the targeting of key signaling pathways, specifically the downregulation of the PhosphatidylInositol 3-Kinase (PI3K)/Protein Kinase B (Akt) and MAPK pathways, which are critical for cell proliferation and survival [13]. The phenolic hydroxyl groups of xanthoxylin also possess free radical-scavenging activity, thereby reducing cellular oxidative stress, which may hold potential applications in the prevention and treatment of various diseases [14,15]. Based on the pharmacodynamic properties of xanthoxylin and the pathophysiological mechanisms underlying ALF, it is hypothesized that xanthoxylin may influence liver failure through modulation of oxidative stress markers, inhibition of inflammatory processes, and the NF-κB signaling pathway.

This study utilized a rat model of liver failure induced by D-galactosamine (D-GalN) combined with lipopolysaccharide (LPS) to investigate the hepatoprotective mechanism of xanthoxylin. The mechanism of action was elucidated through the Bax/Bcl-2 and p38 MAPK/NF-κB signaling pathways, alongside an integrative non-targeted liver metabolomics analysis to identify differential metabolites and associated metabolic pathways. Notably, unlike previous studies that focused on single pathways, this work innovatively integrates pharmacodynamic indices with untargeted metabolomics and correlation network analysis, revealing that xanthoxylin simultaneously modulates amino acid metabolism, the TCA cycle, and NF-κB signaling. This multi-omics perspective provides a systems-level understanding of its hepatoprotective effects, offering valuable experimental data for the prevention and treatment of liver failure.

## 2. Results

### 2.1. Oxidative Stress Indexes and Inflammatory Factors of Liver Tissue

**Figure 1:**
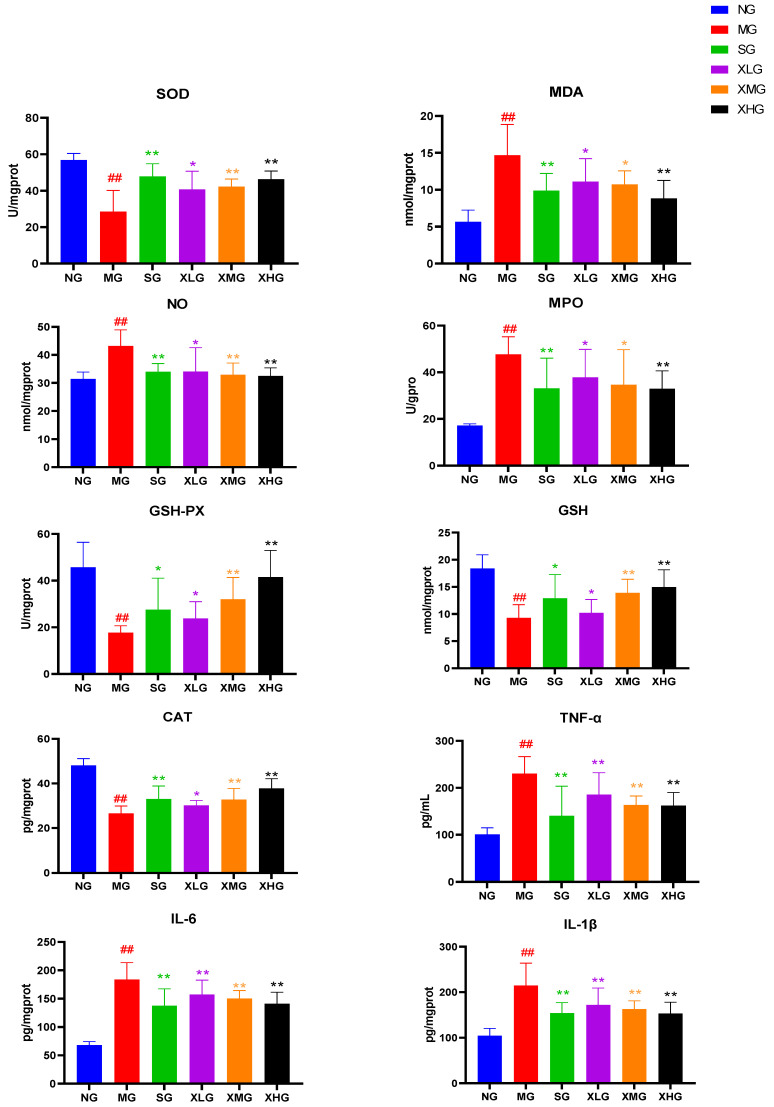
Effects of xanthoxylin on the liver tissue level of 10 factors in acute liver failure rats induced by D-GalN/LPS ($\bar{x}$ *± s*, *n* = 10). Note: Compared with NG, ## *p* < 0.01. Compared with MG, * *p* < 0.05, ** *p* < 0.01.

Compared with the NG group, the levels of superoxide dismutase (SOD), glutathione (GSH), glutathione peroxidase (GSH-Px), and catalase (CAT) in the liver tissue of the MG group were significantly decreased (*p* < 0.01), while the levels of nitric oxide (NO), myeloperoxidase (MPO), malondialdehyde (MDA), TNF-α, IL-6, and IL-1β were significantly increased (*p* < 0.01). In comparison to the MG group, the levels of SOD, GSH, GSH-Px, and CAT in the liver tissues were markedly increased in the SG, XLG, XMG, and XHG groups (*p* < 0.05 or *p* < 0.01), while the levels of NO, MPO, MDA, TNF-α, IL-6, and IL-1β were markedly decreased (*p* < 0.05 or *p* < 0.01), as shown in Figure 1.

### 2.2. The Expression of IL-1β, IL-6, TNF-α, iNOS, NF-κB, IκBα, and TLR4 mRNA in Liver Tissue

**Figure 2:**
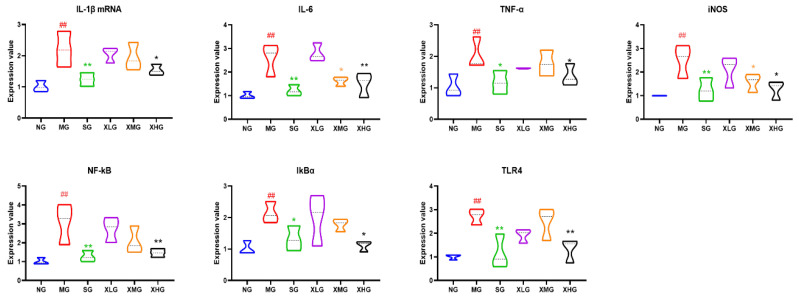
The expression of mRNA in liver tissue (*n* = 3). Note: Compared with NG, ## *p* < 0.01. Compared with MG, * *p* < 0.05, ** *p* < 0.01.

Compared with the NG group, the hepatic mRNA expression of iNOS, IL-1β, IL-6, TNF-α, NF-κB, IκBα, and TLR4 was significantly upregulated in the MG group (*p* < 0.01). Relative to the MG group, the mRNA expression of iNOS, IL-1β, IL-6, TNF-α, NF-κB, IκBα, and TLR4 was downregulated in the SG group (*p* < 0.05 or *p* < 0.01). In the XMG group, the mRNA expression of IL-6 and iNOS was significantly reduced (*p* < 0.05), while in the XHG group, the mRNA expression of iNOS, IL-1β, IL-6, TNF-α, NF-κB, IκBα, and TLR4 was significantly downregulated (*p* < 0.05 or *p* < 0.01), as shown in Figure 2.

### 2.3. The Expression of Bax, Bcl-2, Bcl-xl, and p38 MAPK/NF-κB Signaling Pathway-Related Proteins in Liver Tissue

**Figure 3:**
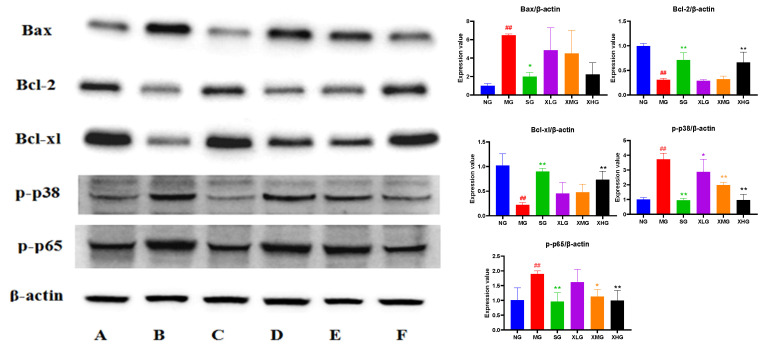
Expression of Bax, Bcl-2, Bcl-xl, p-p38, and p-p65 in rat liver tissue (*n* = 3). A: NG; B: MG; C: SG; D: XLG; E: XMG; F: XHG. Note: Compared with NG, ## *p* < 0.01. Compared with MG, * *p* < 0.05, ** *p* < 0.01.

Compared with the NG group, the hepatic protein expression of Bax, p-p38, and p-p65 was upregulated in the MG group (*p* < 0.01), and the expression of Bcl-2 and Bcl-xl protein was downregulated (*p* < 0.01). In comparison to the MG group, the protein expression of Bax, p-p38, and p-p65 was significantly decreased in the SG group (*p* < 0.01), and the expression of Bcl-2 and Bcl-xl was significantly increased (*p* < 0.05). For the xanthoxylin-treated groups: the hepatic p-p38 protein expression was downregulated in the XLG group (*p* < 0.05); the expression of p-p38 and p-p65 proteins was downregulated in the XMG group (*p* < 0.05 or *p* < 0.01); and in the XHG group, the expression of Bcl-2 and Bcl-xl proteins was upregulated (*p* < 0.01), while the expression of p-p38 and p-p65 proteins was downregulated (*p* < 0.01), as shown in Figure 3.

### 2.4. Metabolomics Analysis

#### 2.4.1. Principal Component Analysis (PCA)

**Figure 4:**
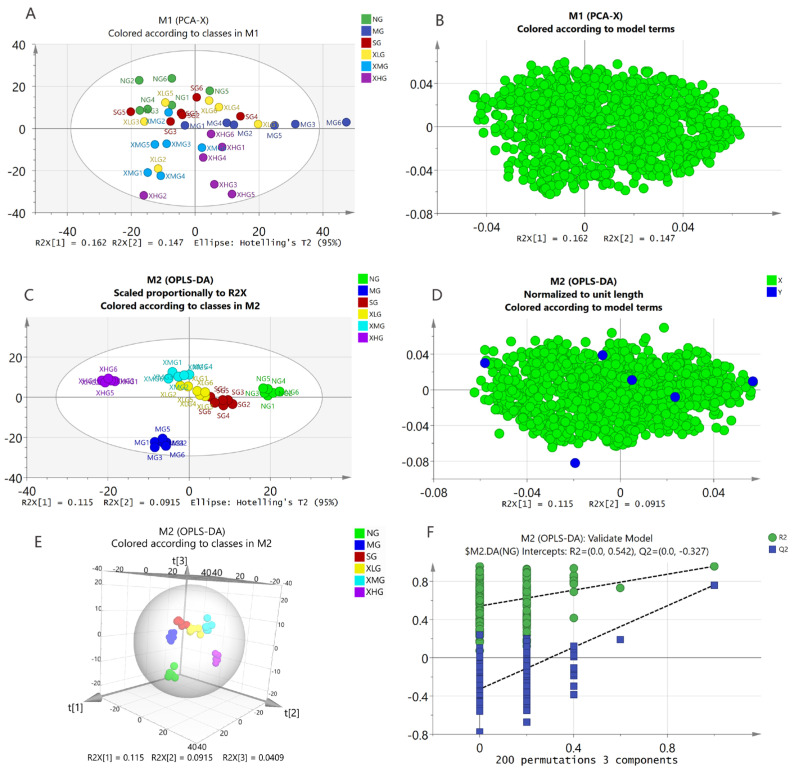
PCA and OPLS-DA analysis results. (**A**) PCA score scatter plot; (**B**) PCA Load diagram; (**C**) OPLS-DA scores scatter plot; (**D**) OPLS-DA Load diagram; (**E**) OPLS-DA 3D plot; (**F**) random displacement test (*n* = 200) and the explanatory rate of OPLS-DA.

Principal component analysis (PCA), a multivariate analysis method, was used to visually evaluate similarities and differences among the six groups. As shown in Figure 4A, the separation between the six groups was not distinct, suggesting that xanthoxylin may regulate the perturbed endogenous metabolites in the rat liver to normal levels. Figure 4B presents the loading plot (also known as the correlation plot), which groups highly correlated variables together, and is referred to as the correlation plot, where highly correlated variables are clustered together, and negatively correlated variables are distributed at both ends of the line passing through the origin. R^2^X(1) and R^2^X(2) are the corresponding contribution rates of PC1 and PC2. The values R^2^X(1) = 0.162 and R^2^X(2) = 0.147 indicated that the contribution of these two principal components was relatively modest.

#### 2.4.2. Orthogonal Partial Least Squares Discriminant Analysis (OPLS-DA)

The OPLS-DA score scatter plot was shown in Figure 4C, and the 3D score plot was shown in Figure 4E. The results showed that the MG was well separated from the NG, SG, XLG, XMG, and XHG groups, indicating that D-GalN/LPS-induced liver failure significantly changed the level of endogenous metabolites in the liver of rats. The OPLS-DA model was used to compare the interpretation rate and prediction ability between the MG group and each of the other groups. For the MG and NG groups, the model yielded Q^2^ = 0.837, R^2^X = 0.489, and R^2^Y = 0.999; for the MG and SG groups, the results were Q^2^ = 0.796, R^2^X = 0.692, R^2^Y = 1.00; the results of MG and XLG groups, Q^2^ = 0.671, R^2^X = 0.722, and R^2^Y = 1.00; for the MG and XMG groups, Q^2^ = 0.719, R^2^X = 0.386, R^2^Y = 0.983; for the MG and XHG groups, Q^2^ = 0.866, R^2^X = 0.710, and R^2^Y = 1.00. All these group comparisons exhibited high predictability, as evidenced by the Q^2^ values. As shown in Figure 4D, R^2^X(1) = 0.115 and R^2^X(2) = 0.0915, indicating that the contribution of these two principal components was relatively low. To validate the OPLS-DA model, 200 random permutation tests were performed on the data. A *p* < 0.05 indicated that the model was valid and reliable, as presented in Figure 4F.

#### 2.4.3. Identification and Change Trend of Potential Biomarkers

Based on the fold change (FC) and variable importance in projection (VIP) values from the comparison between the MG and NG groups, the screening criteria for significantly differential metabolites were established: FC ≥ 2 or ≤0.5 and VIP ≥ 1 (derived from the OPLS-DA model). A total of 41 differential metabolites were screened as biomarkers for liver failure models (*p* < 0.05). After the success of the liver failure model, the levels of 23 differential metabolites increased, while 18 were decreased. After xanthoxylin treatment, all differential metabolites except 2,6-Di-tert-butyl-4-(hydroxymethyl)phenol were restored to normal levels, suggesting that these 40 differential metabolites play key roles in anti-liver failure. The detailed results were shown in Table 1.

#### 2.4.4. Metabolic Pathway Analysis of Potential Biomarkers

**Figure 5:**
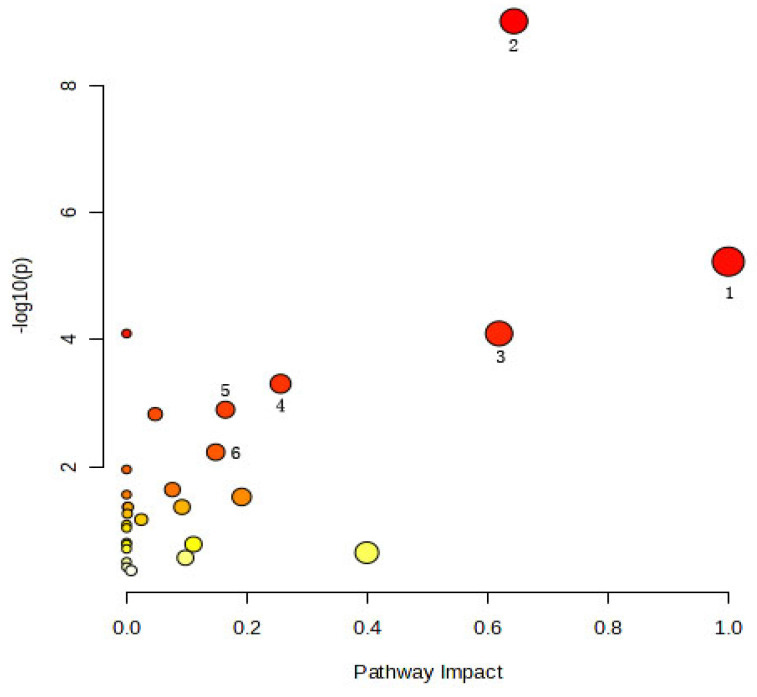
Anti-liver failure metabolic pathway related to xanthoxylin. Note: Colors range from yellow to red based on the −log10(*p*-value); the deeper the color, the more significant the enrichment.1. Phenylalanine, tyrosine, and tryptophan biosynthesis; 2. Glycine, serine, and threonine metabolism; 3. Phenylalanine metabolism; 4. Cysteine and methionine metabolism; 5. Tyrosine metabolism; 6. Glyoxylate and dicarboxylate metabolism.

To identify the metabolic pathways involved in the hepatoprotective mechanism of xanthoxylin, KEGG pathway enrichment analysis was performed on the 41 differential metabolites. The results revealed six major metabolic pathways implicated in the mechanism of xanthoxylin against liver failure, including phenylalanine, tyrosine, and tryptophan biosynthesis; glycine, serine, and threonine metabolism; phenylalanine metabolism; cysteine and methionine metabolism; tyrosine metabolism; and glyoxylate and dicarboxylate metabolism. These pathways are closely related to the anti-liver failure mechanism of xanthoxylin (Figure 5).

### 2.5. Correlation Analysis Between Biomarkers and Pharmacodynamic Indicators

Pearson correlation analysis was performed to evaluate the associations between 41 differential metabolites and 10 pharmacodynamic parameters, including SOD, NO, MPO, MDA, GSH-Px, GSH, CAT, TNF-α, IL-6, and 1L-β. The results revealed the following correlations (all *p* < 0.05): 8 differential metabolites exhibited a positive correlation with SOD, while 8 showed a negative correlation; 20 metabolites were positively correlated with NO, and 12 were negatively correlated; 13 metabolites displayed a positive correlation with MPO, and 6 were negatively correlated; 13 metabolites were positively correlated with GSH-Px, whereas 19 were negatively correlated; 6 metabolites showed a positive correlation with GSH, and 13 were negatively correlated; 13 metabolites were positively correlated with TNF-α, and 8 were negatively correlated; 13 metabolites exhibited a positive correlation with IL-6, and 8 were negatively correlated; 8 metabolites were positively correlated with 1L-β, and 8 were negatively correlated. Notably, 20 differential metabolites, including isonicotinic acid, nicotinic acid, androstenedione, FFA (16:2), carnitine C6:1, pinolenic acid, nootkatone, FFA (18:3), MG (22:5/0:0/0:0), MG (0:0/22:5/0:0), L-asparagine, Leu-Ala, L-glutamine, Met-Tyr, Met-Met, Met-Phe, Phe-Asn, Phe-Tyr, 15(S)-HETrE, and (±)16-HETE, demonstrated robust correlations with all 10 pharmacodynamic parameters (*p* < 0.05).

**Figure 6:**
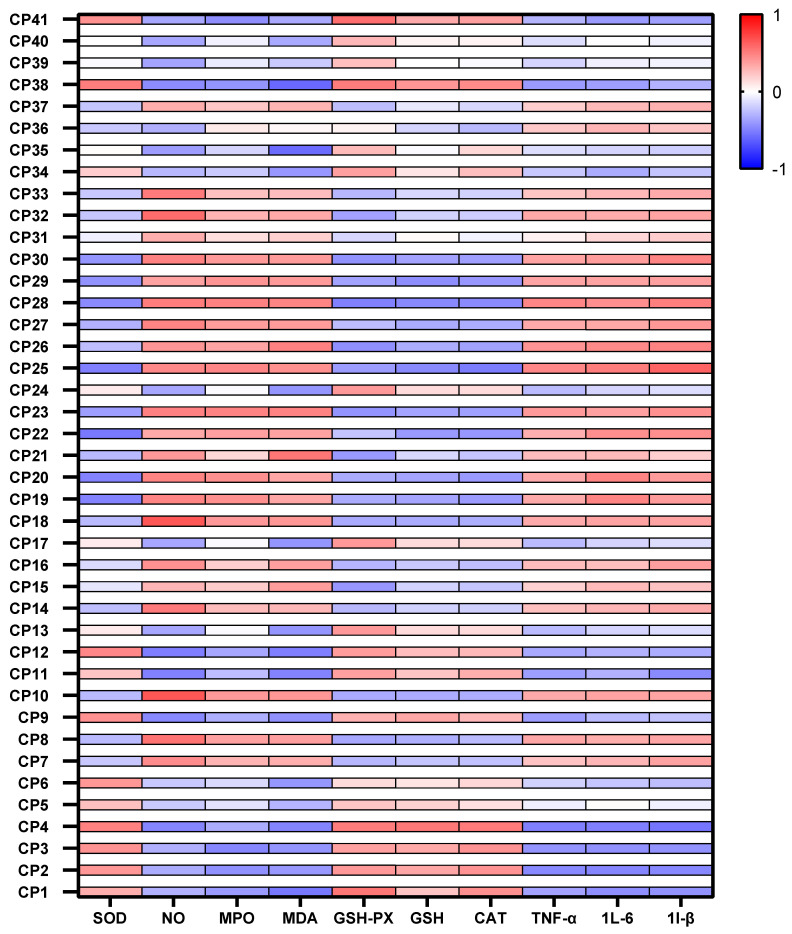
Correlation analysis between 41 differential metabolites and 10 pharmacodynamic indexes. Note: “red” positive correlation; “blue” negative correlation.

These correlations suggest that these 20 differential metabolites may serve as candidate biomarkers associated with hepatoprotection against liver failure. However, as correlation does not imply causation, further mechanistic studies (e.g., gene knockdown, pharmacological intervention, or pathway validation) are required to determine whether these metabolites are functionally involved in the hepatoprotective effects of xanthoxylin or are merely secondary correlates of changes in oxidative stress and inflammatory markers. The detailed results were shown in Figure 6.

## 3. Discussion

Oxidative stress significantly contributes to ALF by generating ROS, which trigger cellular death and inflammation. In a study on D-GalN/LPS-induced liver toxicity, oxidative stress was shown to increase glycogen synthase kinase 3β activity, exacerbating liver damage through increased ROS production and activation of apoptotic pathways [16]. In the present study, xanthoxylin treatment effectively mitigated oxidative stress, as evidenced by increased levels of SOD, GSH, GSH-Px, and CAT, alongside reduced levels of NO, MPO, and MDA. These findings underscore the potential of xanthoxylin in ALF therapy.

The balance between pro-apoptotic (Bax) and anti-apoptotic (Bcl-2) proteins governs cellular survival or death. Activation of caspase-3, a key executioner caspase, initiates the apoptotic cascade. In animal models of virus-induced ALF [17], upregulation of Bax and downregulation of Bcl-2 trigger intracellular apoptotic signaling, and activation of caspase-3 is closely related to the role of Bcl-2 family proteins, which play a key role in virus-induced cell death. In this study, the apoptosis rate of rat liver tissue cells was reduced in the XLG, XMG, and XHG groups. Western blot (WB) analysis revealed that successful ALF modeling led to upregulated Bax protein expression and downregulated Bcl-2 and Bcl-xl protein expression in liver tissues. In contrast, xanthoxylin treatment led to a significant decrease in Bax levels and a marked increase in Bcl-2 and Bcl-xl expression. These observations indicate that Bcl-2 and Bax not only participate in intracellular apoptotic mechanisms but may also become important targets for intervening in acute liver failure. We acknowledge that targeted validation may miss novel pathways. Future unbiased RNA-seq and phosphoproteomics are warranted.

TNF-α, IL-1β, and IL-6 can activate the NF-κB signaling pathway, which is a master regulator of inflammation. Upon activation, the NF-κB pathway drives the transcription of pro-inflammatory genes, thereby amplifying the inflammatory cascade. A study on copper-induced hepatic inflammation demonstrated that oxidative stress triggers the activation of both NF-κB and MAPK pathways, leading to enhanced expression of pro-inflammatory cytokines and liver dysfunction [18]. Furthermore, the study on the protective effect of geraniol in spinal cord injury illustrated that modulation of the p38 MAPK pathway can suppress inflammation and oxidative stress, highlighting potential therapeutic strategies for conditions like ALF [19]. In the present study, xanthoxylin significantly reduced the levels of TNF-α, IL-6, and IL-1β in liver tissue, and significantly inhibited the mRNA expression of NOS, IL-1β, IL-6, TNF-α, NF-κB, IκB-α, and TLR4. WB analysis showed that the expression of p-p65 protein was upregulated in ALF, while the treatment with xanthoxylin led to a significant decrease in p-p65 levels. This suggests that the inducers are recognized by TLR4, which activates IκB-α and promotes its ubiquitination. The ubiquitinated IκB-α then dissociates from the NF-κB dimer, allowing p65 to translocate to the nucleus and induce the expression of pro-inflammatory cytokines (TNF-α, IL-6, and IL-1β), thereby initiating the inflammatory response. Xanthoxylin decreased the level of inflammatory factors by inhibiting the protein levels of IκB-α, p-NF-κB p65, and NF-κB p65 in the NF-κB pathway. Additionally, the expression level of p-p38 protein was downregulated, suggesting that xanthoxylin inhibits p38 MAPK.

The metabolic changes in ALF, especially the changes in the glycolytic pathway and tricarboxylic acid (TCA) cycle, significantly affect the immune response and energy metabolism during ALF. Impaired mitochondrial function is closely related to changes in serum metabolites such as serine and glycine, which are essential for redox balance and immune system regulation [20]. Mahuang Decoction exerts hepatoprotective effects against ALF by modulating the TCA cycle and amino acid metabolism [21]. Collectively, these studies underscore the value of metabolomic approaches in delineating dysfunctional metabolic pathways in ALF, thereby identifying potential targets for therapeutic intervention. In the present study, the changes in metabolites were monitored in the liver. It was found that the levels of 41 differential metabolites were affected in the MG group: 23 were upregulated and 18 were downregulated. After xanthoxylin intervention, 40 of these metabolites were restored to normal levels. Pearson correlation analysis results showed that 41 differential metabolites and 10 pharmacodynamic indexes showed a certain correlation. Notably, seven metabolites, isonicotinic acid, nicotinic acid, androstenedione, carnitine C6:1, nootkatone, 15(S)-HETrE, and (±)16-HETE, were upregulated by xanthoxylin, showing positive correlations with SOD, GSH-Px, GSH, and negative correlations with NO, MPO, TNF-α, IL-6, and IL-β. Thirteen other metabolites, FFA (16:2), pinolenic acid, FFA (18:3), MG (22:5/0:0/0:0), MG (0:0/22:5/0:0), L-asparagine, Leu-Ala, L-glutamine, Met-Tyr, Met-Met, Met-Phe, Phe-Asn, and Phe-Tyr, were downregulated by xanthoxylin, displaying negative correlations with SOD, GSH-Px, and GSH, and positive correlations with NO, MPO, TNF-α, IL-6, and IL-β. Among the 20 candidate metabolite signatures identified, three classes warrant specific biological interpretation. L-Glutamine serves dual roles as a TCA cycle anaplerotic substrate and a direct precursor for glutathione synthesis, both of which are critical for mitochondrial function and antioxidant defense in liver failure [22,23]. In our study, its elevation in ALF and normalization by xanthoxylin, coupled with correlations to SOD/GSH-Px, suggests that xanthoxylin may redirect glutamine metabolism from pathological accumulation toward productive antioxidant pathways. Phenylalanine and tyrosine are aromatic amino acids whose catabolism generates reactive oxygen species and is impaired by oxidative inactivation of phenylalanine hydroxylase [24]. The correlation of their dipeptides with pro-inflammatory cytokines indicates that xanthoxylin may restore amino acid homeostasis. Polyunsaturated fatty acids (FFA 16:2, 18:3) are highly susceptible to lipid peroxidation, generating aldehydes that activate TLR4/NF-κB signaling [25]. Their strong positive correlation with MDA and TNF-α and negative correlation with GSH-Px support the interpretation that xanthoxylin limits substrate availability for oxidative membrane damage.

**Figure 7:**
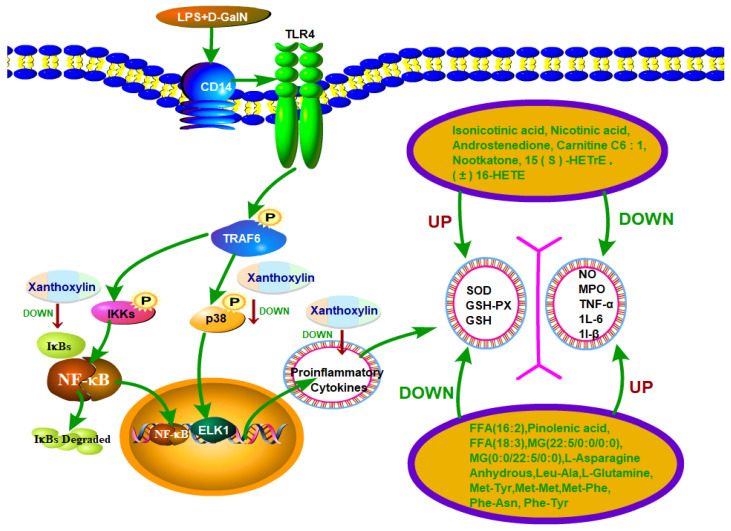
Mechanism diagram of the anti-liver failure effect of xanthoxylin.

In addition, KEGG pathway enrichment analysis revealed six major metabolic pathways enriched by the 41 differential metabolites: phenylalanine, tyrosine, and tryptophan biosynthesis; glycine, serine, and threonine metabolism; phenylalanine metabolism; cysteine and methionine metabolism; tyrosine metabolism; glyoxylate and dicarboxylate metabolism. These pathways are amino acid and carboxylic acid metabolic pathways and belong to the glycolytic pathway and the TCA cycle, which are consistent with the results reported in the literature. Together with the metabolite-level evidence described above, these observations suggest that xanthoxylin exerts its anti-liver failure effects, at least in part, by regulating the glycolytic pathway and the TCA cycle (Figure 7).

The 20 metabolite signatures identified here, particularly those involved in the TCA cycle and amino acid metabolism, hold potential as candidate biomarkers for ALF severity or treatment response. Future clinical studies should validate their utility in patient serum and their correlation with MELD scores.

Several limitations should be acknowledged. First, our targeted validation approach focused on predefined inflammatory and apoptotic pathways; unbiased transcriptomic or proteomic screens (e.g., RNA-seq, phosphoproteomics) could reveal additional xanthoxylin targets. Second, correlation analysis alone cannot establish causal mechanisms—follow-up studies using metabolite supplementation, genetic knockdown, or isotope tracing are needed. Third, the exploratory nature of our metabolomics analysis (no FDR correction) may increase the risk of type I errors; findings require validation in independent cohorts. Fourth, our results are derived from a rat model; cross-species validation in human samples is essential for translational relevance.

## 4. Materials and Methods

### 4.1. Reagents and Instruments

Xanthoxylin (purity 98.23%, No. Lf1129186229, Shanghai Haohong Biomedical Technology Co., Ltd., Shanghai, China). Silymarin (purity 98.95%, No. MUST-23030607, Chengdu Manster Biotechnology Co., Ltd., Chengdu, China). D-GalN (No. 20230420, Shandong Xiya Chemical Co., Ltd., Zaozhuang, China). BCA (No. P0009, Shanghai Biyuntian Biotechnology Co., Ltd., Shanghai, China). SOD, GSH, GSH-PX, CAT, NO, MPO, MDA, TNF-α, IL-1β, IL-6, CRP (No. FU076J0R1206, No. FU04L80V4953, No. FU13608H7791, No. FU01XHJ83855, No. FU16ZDZ24356, No. FU128Z6B4508, No. FU05V6J88079, No. AK096X601931, No. FU094BNB7777, No. FU084N6F4651, No. AK00V0889630, Elabscience Biotechnology Co., Ltd., Wuhan, China). Molpure^®^ Cell/Tissue Total RNA Kit (No. 19221ES50, YEASEN, Shanghai, China). PrimeScript RT reagent Kit and TB Green TM Premix Ex TaqTM II (Tli RNaseH Plus) (No. RR047A, No. RR820A, BaoRiYi Biotechnology Co., Ltd., Shiga Prefecture, Japan). LPS, Western and IP cell lysate, Protease inhibitor universal type 100×, Phosphatase inhibitor 100×, SDS-PAGE Protein loading buffer (5×) and Marker (No. 1031Q0312, No. BL509A, No. BL612A, No. BL615A, No. BL502A, No. BL712A, Beijing Solaibao Technology Co., Ltd., Beijing, China). Bis-Tris 10% high-resolution prefabricated adhesive, Tris-Mops (No. 36266ES10, No. 36271ES05, yeasen, Shanghai, China). Immobilon-PSQ PVDF (No. ISEQ00010, Sigma Aldrich, Taufkirchen, Germany). glycine, Primary antibody and secondary antibody eluent (No. GC304019, No. G2016, servicebio, Wuhan, China). ‘Torchlight’ Hypersensitive ECL Western HRP Substrate (No. 17046, zen-bio, Durham, NC, USA). HRP Goat Anti-Mouse IgG (H+L), β-actin, Bax (No. AS003, abclonal, No. AC026, abclonal, No. A19684, abclonal, Wuhan, China). Bcl-2, Bcl-xl, p-p38 (No. 68103-1-Ig, No. 10783-1-AP, No. 28796-1-AP, Proteintech, Wuhan, China). Goat Anti-Rabbit IgG (H+L) HRP, p-p65 (No. S0001, No. AF2006, affbiotech, Liyang, China). LC-MS grade acetonitrile (ACN) was purchased from Fisher Scientific (Loughborough, UK). Formicacid was obtained from TCI (Shanghai, China). Ammonium formate was obtained from Sigma-Aldrich (Shanghai, China). Ultrapure water was generated using a Milli-Q system (Millipore, Bedford, MA, USA).

The LC analysis was performed on a Vanquish UHPLC System (Thermo Fisher Scientific, Waltham, MA, USA). Mass spectrometric detection of metabolites was performed on Orbitrap Exploris 120 (Thermo Fisher Scientific, USA) with ESI ion source. Freeze centrifuge (5810R model, Eibend Company, Plauen, Germany). Full wavelength enzyme-linked immunosorbent assay (Epoch2 model, Bio Tek Company, Norcross, GA, USA). Real-time fluorescence quantification (RT-PCR) instrument (QuantStudio TM3, Thermo Fisher Instruments Co., Ltd., Waltham, MA, USA). Fluorescence image analysis system (5200 Multi, Tanon, Shanghai, China).

### 4.2. Experimental Animals and Modeling of Acute Liver Failure

A rat model of liver failure was induced by D-GalN combined with LPS. Sixty SPF-grade male Sprague-Dawley (SD) rats, weighing 140–170 g, were purchased from Hunan SJA Laboratory Animal Co., Ltd. (Changsha, China), with the production license number SCXK (Xiang) 2019-0004 and the quality certificate number 430727231101981334. All rats were housed at the Experimental Animal Center of Guangxi University of Chinese Medicine, which holds the experimental animal use license number SYXK (Gui) 2019-0001. Sample size was calculated using Microsoft Excel software(2016) based on a pilot study: with α = 0.05, power = 0.80, and an expected 30% difference in SOD levels between groups, a minimum of 8 rats per group was required. Accounting for a 20% dropout rate, we used *n* = 10 per group.

Rats were stratified by body weight and randomly assigned to six groups (*n* = 10 each) using a random number table generated by Microsoft Excel software. The 60 SD rats were randomly divided into 6 groups: normal group (NG, *n* = 10, 10 mL/kg 0.5% carboxymethylcellulose sodium [CMC-Na] intragastrically [i.g.]); model group (MG, *n* = 10, 10 mL/kg 0.5% CMC-Na i.g.); silymarin group (SG, *n* = 10, 50 mg/kg silymarin i.g.); low-dose xanthoxylin group (XLG, *n* = 10, 60 mg/kg xanthoxylin i.g.); medium-dose xanthoxylin group (XMG, *n* = 10, 120 mg/kg xanthoxylin i.g.); and high-dose xanthoxylin group (XHG, *n* = 10, 240 mg/kg xanthoxylin i.g.) [15]. Rats were administered the respective treatments once daily for 14 consecutive days. On Day 13, the rats were fasted for 14–16 h without water deprivation. At 1 h after the last administration, the rats in NG were intraperitoneally injected with normal saline at 2 mL/kg, and the MG, SG, XLG, XMG, and XHG rats were intraperitoneally injected with 2 mL/kg of a D-GalN + LPS mixture (D-GalN 400 mg/kg; LPS 30 μg/kg).

Blinding was implemented throughout the experiment: the investigator responsible for drug administration, sample collection, biochemical measurements, Western blot quantification, and metabolomic data processing was blinded to group allocation. All samples were coded, and the code was broken only after data analysis was completed.

Exclusion criteria: After 7 days of adaptive feeding, the average body weight of rats was > or <20%. There are no animals that meet the above exclusion criteria.

All animal studies were approved by the Ethics Committee of Guangxi University of Chinese Medicine (approval number: DW20231016-200).

### 4.3. Detection of Oxidative Factors and Inflammatory Factors in Liver Tissue by ELISA

Approximately 200 mg of liver tissue was harvested, and iced-cold normal saline was added at a liver weight:volume of 1:9 (*w*/*v*). The tissue was minced and homogenized to prepare a 10% liver tissue homogenate using a homogenizer. After centrifugation at 3000 rpm for 10 min, the supernatant was collected and sub-packed in a 200 μL EP tube. The levels of SOD, MDA, NO, MPO, GSH-Px, GSH, CAT, TNF-α, IL-6, and IL-1β were determined by ELISA.

### 4.4. Detection of Inflammatory Factor mRNA Expression Level in Liver Tissue by RT-qPCR

Ten to twenty milligrams (10–20 mg) of liver tissue was homogenized using a low-temperature grinder, from which total RNA was extracted. The concentration and purity of RNA were detected by an ultra-micro spectrophotometer. cDNA was prepared by reverse transcription and amplified by real-time quantitative PCR (RT-qPCR). The primer sequences were designed and synthesized by Shanghai Shenggong Bioengineering Technology Service Co., Ltd. (Shanghai, China), as follows: β-actin (forward: 5′-GGGAAATCGTGCGTGACATT-3′; backward: 5′-GCGGCAGTGGCCATCTC-3′), IKBα (forward: 5′-ACTTGGTGACTTTGGGTGCTGATG-3′; backward:5′-CCACACTTCAACAGGAGCGAGAC-3′), IL-1β (forward: 5′-TCTCACAGCAGCATCTCGACAAG-3′; backward: 5′-CCACGGGCAAGACATAGGTAGC-3′), IL-6 (forward: 5′-GCCTTCTTGGGACTGATGTTGTTG-3′; backward: 5′-GTCTGTTGTGGGTGGTATCCTCTG-3′), iNOS (forward: 5′-GGAAGAGACGCACAGGCAGAG-3′; backward: 5′-CAGCAGGCACACGCAATGATG-3′), NF-KB (forward: 5′-ATGGCTTCTATGAGGCTGAACTCTG-3′;backward:5′-TTGCTCCAGGTCTCGCTTCTTC-3′), TLR4 (forward: 5′-AGAATGAGGACTGGGTGAGAAACG-3′; backward: 5′-CTGGATGATGTTGGCAGCAATGG-3′), TNF-α (forward: 5′-CCGAGATGTGGAACTGGCAGAG-3′; backward: 5′-CCACGAGCAGGAATGAGAAGAGG-3′). The total volume of the PCR reaction system was 20 μL, consisting of 10.0 μL 2× Real PCR Easy^TM^ Mix-SYBR, 0.8 μL of Forward Primer (10 μM), 0.8 μL of Reverse Primer (10 μM), 2.0 μL of template cDNA, and RNase-free water to bring the final volume to 20 μL. The PCR amplification conditions were pre-denaturation at 95 °C for 30 s, denaturation for 5 s, annealing/extension at 55 °C for 30 s, and full extension at 72 °C for 30 s, a total of 45 cycles. The relative expression levels of IL-1β, IL-6, TNF-α, iNOS, NF-κB, IκBα, and TLR4 mRNA were calculated by the 2^−ΔΔCT^ method using β-actin as an internal reference.

### 4.5. Western Blot Analysis of Liver

Approximately 300 mg of liver tissue was harvested, and 10 times the amount of RIPA lysis buffer containing protease inhibitors was added. The tissue was homogenized using a high-speed, low-temperature grinder (−20 °C, grinding 4 times, 60 s each) and then incubated on ice for 30 min with continuous shaking (5 min/time) to ensure complete lysis. After centrifugation at 12,000 rpm for 10 min at 4 °C, the supernatant was collected to obtain the total protein solution. Protein concentration was determined by the BCA method. Protein samples were mixed with reducing protein loading buffer at a ratio of 4:1, followed by heat denaturation at 95 °C for 15 min. Sodium dodecyl sulfate–polyacrylamide gel electrophoresis (SDS-PAGE) was performed at 100 V until the bromophenol blue front reached the bottom of the gel, after which electrophoresis was terminated. Proteins were transferred onto polyvinylidene difluoride (PVDF) membranes, which were then blocked with 5% skim milk diluted in TBST buffer for 2 h at room temperature. The membranes were incubated overnight at 4 °C with primary antibodies against Bax, Bcl-2, Bcl-xl, p38, p-p38, p65, and p-p65 on a shaker. The membranes were rapidly washed with TBST in the dark on a decolorizing shaker 3 times, 5 min/time. The secondary antibody (HRP-goat anti-rabbit IgG 1:5000) was incubated at room temperature for 2 h, and the membranes were quickly washed with TBST in the dark on a decolorizing shaker 3 times, 10 min/time, followed by chemiluminescence development and fixation, and the membranes were scanned and archived. The strips were imaged using the Tanon Fluorescence Image Analysis System (Version 2.0). Gel-Pro Analyzer 4.0 software was used to scan the exposure results. The relative protein expression levels of Bax, Bcl-2, Bcl-xl, p38, p-p38, p65, and p-p65 were calculated with β-actin as the internal reference.

### 4.6. Statistical Analysis

GraphPad Prism 8.0 and IBM SPSS 20.0 software were used for graph generation and data analysis. All data are presented as the mean ± standard error of the mean (SEM). Statistical significance was determined by Student’s *t*-tests (two-tailed) for two groups. *p* < 0.05 or column with different lowercase letters was used to indicate statistical significance. All experiments were performed with independent biological replicates (*n* = 10 per group for ELISA; *n* = 3 per group for RT-qPCR and Western blot). Each biological replicate was measured in technical triplicate.

### 4.7. UPLC-MS Analysis

#### 4.7.1. Sample Preparation

A total of 20 mg (±1 mg) of liver tissue samples was taken into a centrifuge tube. Samples were homogenized using a ball mill (30 Hz) for 20 s, followed by centrifugation at 3000 rpm for 30 s at 4 °C. Subsequently, 400 μL of 70% methanol-water solution containing an internal standard was added, and the mixture was vortexed at 2500 rpm for 5 min before being allowed to stand on ice for 15 min. After centrifugation at 12,000 rpm for 10 min at 4 °C, 300 μL of the supernatant was collected and stored at −20 °C for 30 min. Following an additional centrifugation at 12,000 rpm for 3 min, 200 μL of the supernatant was removed for analysis.

#### 4.7.2. Chromatographic Conditions

Chromatography was carried out with an ACQUITY UPLC ^®^ HSS T3 (2.1 × 100 mm, 1.8 µm) (Waters, Milford, MA, USA). The column maintained at 40 °C. The flow rate and injection volume were set at 0.3 mL/min and 5 μL, respectively. For LC-ESI (+)-MS analysis, the mobile phases consisted of (B2) 0.1% formic acid in acetonitrile (*v*/*v*) and (A2) 0.1% formic acid in water (*v*/*v*). Separation was conducted under the following gradient: 0~1 min, 10% B2; 1~5 min, 10%~98% B2; 5~6.5 min, 98% B2; 6.5~6.6 min, 98%~10% B2; 6.6~8 min, 10% B2. For LC-ESI (-)-MS analysis, the analytes was carried out with (B3) acetonitrile and (A3) ammonium formate (5 mM). Separation was conducted under the following gradient: 0~1 min, 10% B3; 1~5 min, 10%~98% B3; 5~6.5 min, 98% B3; 6.5~6.6 min, 98%~10% B3; 6.6~8 min, 10% B3.

#### 4.7.3. Mass Spectrum Conditions

Simultaneous MS1 and MS/MS (Full MS-ddMS2 mode, data-dependent MS/MS) acquisition was used. The parameters were as follows: sheath gas pressure, 40 arb; aux gas flow, 10 arb; spray voltage, 3.50 kV and −2.50 kV for ESI(+) and ESI(-), respectively; capillary temperature, 325 °C; MS1 range, *m*/*z* 100–1000; MS1 resolving power, 60,000 FWHM; number of data dependant scans per cycle, 4; MS/MS resolving power, 15,000 FWHM; normalized collision energy, 30%; dynamic exclusion time, automatic.

#### 4.7.4. Data Analysis and Statistics

Raw mass spectrometry data were acquired using an LC-MS/MS system and initially processed with Analyst 1.6.3 software. The generated raw data were converted to mzML format using ProteoWizard (v3.0.8789). Peak extraction, retention time alignment, feature identification, and filtering were performed using the online XCMS (V3.12.0) platform (http://xcmsonline.scripps.edu, accessed on 30 March 2025). A final data matrix comprising retention time, mass-to-charge ratio (*m*/*z*), and peak area was generated.

After data export, peak area normalization was performed using Excel. Missing values were imputed using half of the minimum positive value per feature. Subsequently, the normalized data matrix was imported into the R package MetaboAnalystR 1.0.1 for multivariate statistical analysis. Principal component analysis (PCA) was first applied to visualize overall sample distribution and detect potential outliers. Orthogonal partial least squares discriminant analysis (OPLS-DA) was then performed to maximize the separation between control and model groups as well as between model and treatment groups. Model validity was assessed by R^2^Y and Q^2^ parameters, with 200 permutation tests to avoid overfitting.

Differential metabolites were selected based on the following criteria: variable importance in projection (VIP) ≥ 1 derived from the OPLS-DA model, fold change (FC) ≥ 2 or ≤0.5 (corresponding to upregulation and downregulation, respectively), and Student’s *t*-test *p*-value < 0.05. Metabolite identification was performed by matching *m*/*z* values and retention times against the KEGG Compound database (http://www.kegg.jp/kegg/compound/, accessed on 12 June 2025). Identified metabolites were then mapped to the KEGG PATHWAY database (http://www.kegg.jp/kegg/pathway.html, accessed on 19 June 2025) for pathway enrichment analysis. Significantly enriched metabolic pathways were determined using hypergeometric tests, with *p*-values < 0.05 considered statistically significant.

All metabolomics experiments were performed with independent biological replicates (*n* = 6 per experimental group).

## 5. Conclusions

Xanthoxylin exerts anti-liver failure effects by preventing hepatocyte apoptosis, suppressing the IκB-α/NF-κB signaling pathway, and regulating the glycolytic pathway and the TCA cycle. In summary, xanthoxylin mediates its anti-ALF effects primarily through interconnected mechanisms. First, intricate interactions among oxidative stress markers, inflammatory cytokines, and signaling pathways such as Bax/Bcl-2 and p38 MAPK/IκB-α/NF-κB are central to the pathogenesis of ALF, and xanthoxylin modulates these key interactions to alleviate liver injury. Second, the 20 potential biomarkers (predominantly amino acids, peptides, and fatty acids) are involved in amino acid and carboxylic acid metabolic pathways, with their mechanisms of action likely related to the glycolytic pathway and the TCA cycle. This aligns with the contemporary understanding of multi-component, multi-target, and multi-pathway synergistic effects of modern medicine.

## Figures and Tables

**Table 1:** Potential biomarkers in the liver failure model.

No.	Compounds	Formula	*p*-Value	Log^2^FC	NG vs. MG	MG vs. SG	MG vs. XLG	MG vs. XMG	MG vs. XHG
CP1	Indole-3-carboxylic acid	C_9_H_7_NO_2_	0.0057 **	−1.2069	↓	-	↑	↑	↑
CP2	Isonicotinic acid	C_6_H_5_NO_2_	0.0028 **	−0.4163	↓	-	↑	↑	↑
CP3	Nicotinic Acid	C_6_H_5_NO_2_	0.0028 **	−0.5455	↓	↑	↑	↑	↑
CP4	Androstenedione	C_19_H_26_O_2_	0.0002 ***	−0.8758	↓	↑	↑	↑	↑
CP5	Androstenediol	C_19_H_30_O_2_	0.0119 *	−0.1404	↓	↑	↑	↑	↑
CP6	Dehydroabietic acid	C_20_H_28_O_2_	0.0004 ***	−4.0150	↓	↑	↑	↑	↑
CP7	Thr-Ile	C_10_H_20_N_2_O_4_	0.0024 **	0.8030	↑	-	↓	↓	↓
CP8	FFA(16:2)	C_16_H_28_O_2_	0.0336 *	0.9581	↑	↓	↓	↓	↓
CP9	Carnitine C6:1	C_13_H_23_NO_4_	0.0029 **	−4.2912	↓	-	↑	↑	↑
CP10	Pinolenic acid	C_18_H_30_O_2_	0.0216 *	0.5455	↑	↓	↓	↓	↓
CP11	Norelgestromin	C_21_H_29_NO_2_	0.0012 **	−4.2877	↓	↑	↑	↑	↑
CP12	Nootkatone	C_15_H_22_O	0.0005 ***	−2.8856	↓	↑	↑	↑	↑
CP13	Xylose	C_5_H_10_O_5_	0.0279 *	−0.4809	↓	-	↑	↑	↑
CP14	Phosphatidylethanolamine lyso alkenyl 18:3	C_23_H_44_NO_6_P	0.0151 *	1.2124	↑	↓	↓	↓	↓
CP15	Lys-Met	C_11_H_23_N_3_O_3_S	0.0061 **	0.5433	↑	-	↓	↓	↓
CP16	Hypaphorine	C_14_H_18_N_2_O_2_	0.0107 *	0.6599	↑	-	↓	↓	↓
CP17	Arabinose	C_5_H_10_O_5_	0.0279 *	−0.4809	↓	-	↑	↑	↑
CP18	FFA(18:3)	C_18_H_30_O_2_	0.0216 *	0.5455	↑	↓	↓	↓	↓
CP19	MG(22:5/0:0/0:0)	C_25_H_40_O_4_	0.0027 **	1.8679	↑	↓	↓	↓	↓
CP20	MG(0:0/22:5/0:0)	C_25_H_40_O_4_	0.0027 **	1.8679	↑	↓	↓	↓	↓
CP21	L-Isoserine	C_3_H_7_NO_3_	0.0111 *	0.6924	↑	-	↓	↓	↓
CP22	L-Asparagine Anhydrous	C_4_H_8_N_2_O_3_	0.0002 ***	2.3907	↑	↓	↓	↓	↓
CP23	Leu-Ala	C_9_H_18_N_2_O_3_	0.0026 **	1.1686	↑	↓	↓	↓	↓
CP24	L-lyxose	C_5_H_10_O_5_	0.0279 *	−0.4809	↓	-	↑	↑	↑
CP25	L-Glutamine	C_5_H_10_N_2_O_3_	0.0003 ***	2.5445	↑	↓	↓	↓	↓
CP26	Met-Tyr	C_14_H_20_N_2_O_4_S	0.0057 **	1.1001	↑	↓	↓	↓	↓
CP27	Met-Met	C_10_H_20_N_2_O_3_S_2_	0.0072 **	0.8251	↑	↓	↓	↓	↓
CP28	Met-Phe	C_14_H_20_N_2_O_3_S	0.0009 ***	1.3280	↑	↓	↓	↓	↓
CP29	Phe-Asn	C_13_H_17_N_3_O_4_	0.0078 **	1.1983	↑	↓	↓	↓	↓
CP30	Phe-Tyr	C_18_H_20_N_2_O_4_	0.0027 **	0.9015	↑	↓	↓	↓	↓
CP31	LPC(O-18:2)	C_26_H_52_NO_6_P	0.0216 *	1.2439	↑	-	↓	↓	↓
CP32	LPC(O-16:1)	C_24_H_50_NO_6_P	0.0205 *	1.1855	↑	↓	↓	↓	↓
CP33	AMC Arachidonoyl Amide	C_30_H_39_NO_3_	0.0265 *	1.2694	↑	↓	↓	↓	↓
CP34	3-O-Methyldopa	C_10_H_13_NO_4_	0.0029 **	−0.5433	↓	-	↑	↑	↑
CP35	2-Phenylpropylamine	C_9_H_13_N	0.0194 *	−0.3485	↓	-	↑	↑	↑
CP36	2,6-Di-tert-butyl-4-(hydroxymethyl)phenol	C_15_H_24_O_2_	0.0038 **	0.1768	↑	-	↑	↑	↑
CP37	18β-Glycyrrhetinic acid	C_30_H_46_O_4_	0.0025 **	1.9944	↑	-	↓	↓	↓
CP38	15(S)-HETrE	C_20_H_34_O_3_	0.0004 ***	−0.8230	↓	↑	↑	↑	↑
CP39	11-Cis-Retinol	C_20_H_30_O	0.0342 *	−1.9802	↓	-	↑	↑	↑
CP40	(±)17-HDHA	C_22_H_32_O_3_	0.0494 *	−0.6291	↓	-	↑	↑	↑
CP41	(±)16-HETE	C_20_H_32_O_3_	0.0220 *	−2.1562	↓	↑	↑	↑	↑

*p* value: * *p* < 0.05,** *p* < 0.01, *** *p* < 0.001; NG vs. MG; ↑: level up; ↓: level down.

## Data Availability

The raw data supporting the conclusions of this article will be made available by the authors on request.
